# Characterization and Programming Algorithm of Phase Change Memory Cells for Analog In-Memory Computing

**DOI:** 10.3390/ma14071624

**Published:** 2021-03-26

**Authors:** Alessio Antolini, Eleonora Franchi Scarselli, Antonio Gnudi, Marcella Carissimi, Marco Pasotti, Paolo Romele, Roberto Canegallo

**Affiliations:** 1 Electrical, Electronic and Information Engineering Department “Guglielmo Marconi”, University of Bologna, Viale Risorgimento 2, 40123 Bologna, Italy; eleonora.franchi@unibo.it (E.F.S.); antonio.gnudi@unibo.it (A.G.); 2 STMicroelectronics, 20864 Agrate Brianza, Italy; marcella.carissimi@st.com (M.C.); marco.pasotti@st.com (M.P.); paolo.romele@st.com (P.R.); roberto.canegallo@st.com (R.C.)

**Keywords:** nonvolatile memory (NVM), phase-change memory (PCM), analog in-memory computing (AIMC)

## Abstract

In this paper, a thorough characterization of phase-change memory (PCM) cells was carried out, aimed at evaluating and optimizing their performance as enabling devices for analog in-memory computing (AIMC) applications. Exploiting the features of programming pulses, we discuss strategies to reduce undesired phenomena that afflict PCM cells and are particularly harmful in analog computations, such as low-frequency noise, time drift, and cell-to-cell variability of the conductance. The test vehicle is an embedded PCM (ePCM) provided by STMicroelectronics and designed in 90-nm smart power BCD technology with a Ge-rich Ge-Sb-Te (GST) alloy for automotive applications. On the basis of the results of the characterization of a large number of cells, we propose an iterative algorithm to allow multi-level cell conductance programming, and its performances for AIMC applications are discussed. Results for a group of 512 cells programmed with four different conductance levels are presented, showing an initial conductance spread under 6%, relative current noise less than 9% in most cases, and a relative conductance drift of 15% in the worst case after 14 h from the application of the programming sequence.

## 1. Introduction

Among non-volatile memories (NVMs), phase-change memory (PCM) is a promising technology for both stand-alone and embedded applications. Due to its successful integration in actual CMOS fabrication processes and its high-throughput performance and read/write endurance, PCMs are considered as a valid technology for next-generation NVMs [1]. In particular, embedded PCM (ePCM) guarantees minimum impact on process complexity and on the other integrated components [2,3], including also high-power and high-voltage components [4].

PCM relies on the reversible transition of a chalcogenide material between its crystalline (or SET) and amorphous (or RESET) state. The amorphous phase tends to have high electrical resistivity, while the crystalline phase exhibits a low resistivity, several orders of magnitude lower.

Consequently, PCM can be exploited as an alternative to conventional binary NVMs [1], as their cells can store a digital “0” or a digital “1”. On the other side, due to their great resistance contrast, the change in read current is quite large, opening up the opportunity for the multiple levels needed for multi-level cell (MLC) operations [5]. The intrinsic capability of a memory cell to store multilevel data allows the possibility to encode more than one bit of digital data per cell. On the other hand, MLC storage requires the cell resistance to be programmed and read with higher accuracy with respect to the case of binary storage.

Another relevant key point of PCM technology is its application to analog in-memory computing (AIMC) [5,6,7,8,9], where all the computation is carried out inside the memory chip in an analog way, avoiding digital data to be conveyed between conventional memory and processing units (the so-called “Von Neumann bottleneck”) [8,9]. 

**Figure 1:**
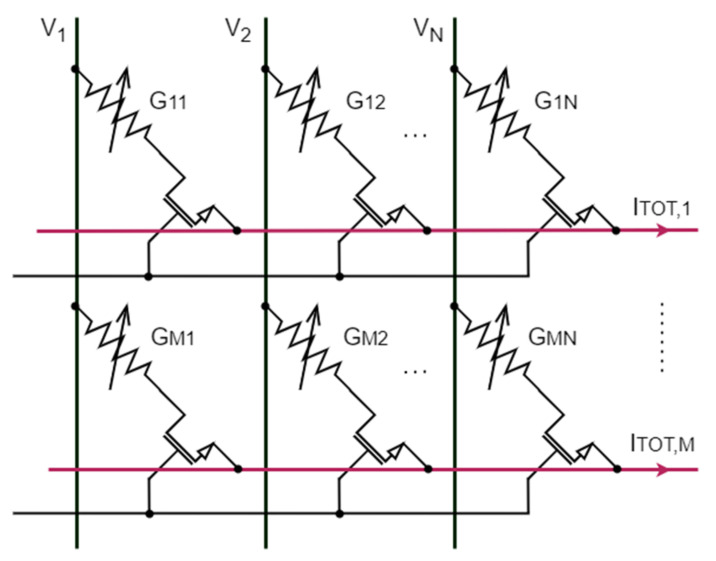
Schematic diagram of a phase-change memory (PCM) array and its use for analog in-memory computing (AIMC).

In this context, PCM technology has been applied to both artificial neural networks [7] and spiking neural networks [10,11]. Some recent review papers are [12,13]. In all these applications, multilevel storage is an attractive peculiarity, as it allows one to easily perform analog multiplications simply exploiting Ohm’s and Kirchhoff’s laws [14,15]. Given a cell with conductance G, a single multiplication is achieved applying to the cell a predefined voltage V, and thus the readout current I satisfies I = GV (Figure 1). If several cells work with their own applied voltage, the sum of their currents *I_TOT_* implements the sum of each product between conductance *G_i_* and voltage *V_i_*, as

(1) $$I_{TOT}=\overset{N}{\underset{i=1}{\sum }}G_{i}V_{i}$$

From this result, it is possible to conceive the whole memory as a conductance matrix G with dimension M × N. Then, applying a voltage vector V to each row, one obtains a matrix-vector multiplication

(2) $$\left(\begin{array}{ccc} G_{11} & \cdots & G_{1N}\\ \vdots & \ddots & \vdots \\ G_{M1} & \cdots & G_{MN} \end{array}\right)\left(\begin{array}{c} V_{1}\\ \vdots \\ V_{N} \end{array}\right)=\left(\begin{array}{c} I_{TOT,1}\\ \vdots \\ I_{TOT,M} \end{array}\right)$$

where *I_TOT_*_,*k*_, with *k =* 1, …, *M*, is the *k*-th total readout current.

0-conductance elements are realized with cells in RESET-state, and non-null conductance elements with cells in a SET-state programmed to have a specified conductance value [16]. Furthermore, PCM cells are characterized by a maximum conductance G^MAX^, which is reached in their full-SET state.

However, from a practical viewpoint, several problems afflict this picture, due to the very nature of PCM cells [17,18,19]:

- Noise: low-frequency (flicker) noise affects cells behavior, as random electron traps are located in the cell lattice, especially in the amorphous region.
- Time drift: cell conductance tends to decrease due to amorphization and relaxation phenomena of the crystal lattice.
- Uncertainty of the initial conductance value: different cells respond differently to the same programming pulses. Moreover, the response of the same cell to subsequent programming cycles shows a large variability. This leads to dispersion and inaccuracy of the conductance levels.

**Figure 2:**
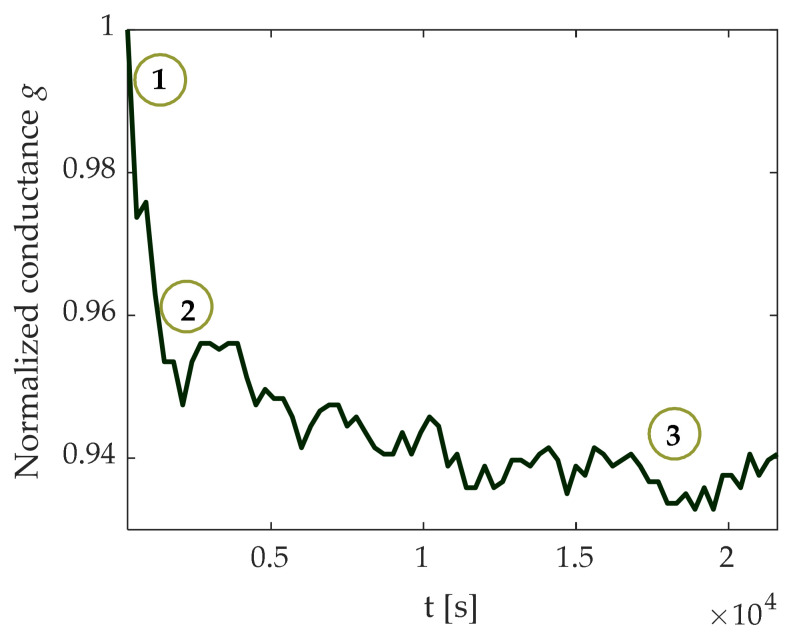
Measured time behavior of a cell normalized conductance showing undesired phenomena: (1) uncertainty of initial value; (2) drift; (3) noise.

To illustrate the above points, the time-behavior of a typical cell is shown in Figure 2, where the measured conductance, normalized to its initial value, is reported. 

In this paper, a non-standard characterization study of PCM cells oriented to AIMC applications is presented, specifically aimed at providing methods and programming strategies able to circumvent, or at least attenuate, the above problems. PCM cells fabricated in STMicroelectronics 90-nm smart-power BCD technology are used as experimental vehicles. As cells in RESET state exhibit a high resistance (in the range of tens of MΩ), their currents are in the range of tens of nanoamperes. When in a SET state, cell resistance falls in the range of tens of kiloohms. Thus, the ratio between a SET state and a RESET state conductance is about 1000, leading to SET cells being more decisive in the weighted sum (1). For this reason, the focus of the paper is on the characterization of cells in SET state and on searching ways to exploit the degrees of freedom of SET pulse parameters (illustrated in Section 2.2) to minimize the undesired phenomena. 

The paper is organized as follows. In Section 2, we illustrate the experimental setup; in Section 3 results in terms of noise, drift, and variability are shown, when single-pulse and multiple-pulse programming se-quences are applied to PCM cells; in Section 4, a programming algorithm is proposed, and its performances are evaluated.

## 2. Material and Methods

### 2.1. PCM Test Chip and Evaluation Board

We performed the experimental activity on an embedded PCM (ePCM) test chip designed and manufactured by STMicroelectronics [20] in 90-nm smart power BCD technology featuring a specifically optimized Ge-rich Ge-Sb-Te (GST) alloy. The chip is intended for digital storage in automotive applications. The ePCM elementary cell is based on an NMOS selector [21] and occupies 0.19 μm^2^ of silicon area. A 256-KB macrocell was included in the test chip in 8 independent instances in order to increase the total number of cells in a single chip. In addition to the 8 ePCM macrocells, the chip also includes a built-in self-test (BIST) block, several configuration registers, a reference generator block, and the circuitry that manages the input–output interface.

A PCM evaluation board (properly designed for testing purposes) was employed and customized. This board allows one to configure current pulses applied to cells, as voltage and current regulators are integrated on the test chip. Furthermore, it is possible to measure the current of single or multiple cells thanks to an analog chip-board interface and a dedicated I-V conversion chain. Every programming or measurement process is achieved with a GUI interface, which is available on a personal computer and customizable. Finally, the evaluation board was equipped with analog to digital converters that allow for the measured current to be stored and elaborated.

### 2.2. Programming Pulses Parameters

**Figure 3:**
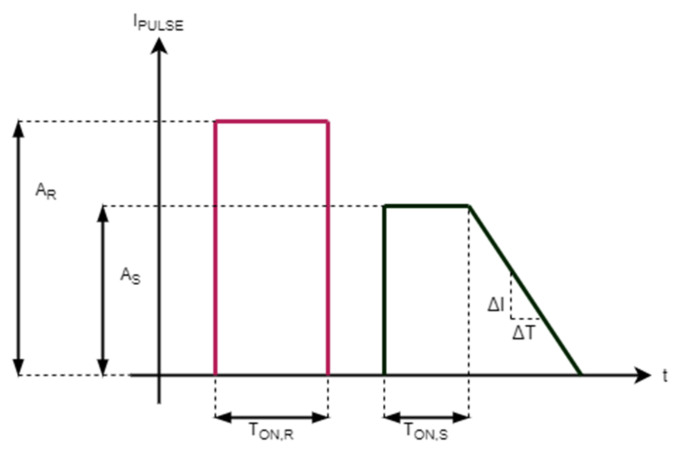
SET and RESET pulses and configurable parameters.

Cell transition between SET state and RESET state is accomplished with the application of a corresponding current pulse [1,22,23], which causes a significant portion of the cell to be heated, in order to modify its internal structure (Figure 3):

- a SET pulse is a trapezoidal current pulse, composed of an initial melting phase, followed by a slow crystallization phase;
- a RESET pulse consists in a higher current flow and it is applied in order to melt the central portion of the cell. The molten material quenches into the amorphous phase, producing a cell in the high-resistance state.

The possibility to set the cell in a wide range of intermediate conductance states is achieved through an adequate control of different configurations of the crystalline and amorphous phases inside the active chalcogenide volume: in other terms, the cell resistance value depends on the shape and the volume of the two phases. The main aim of our set of measurements was to investigate the impact of the different pulse parameters and the associated programming sequences on cells noise, drift, and conductance variability. The pulse parameters that are editable through the evaluation board are indicated in Figure 3, namely,

- the SET pulse can be modulated in amplitude (A_S_), width of the flat portion (T_ON,S_), and decaying slope (ΔI/ΔT);
- the RESET pulse can be modulated in amplitude (A_R_) and width T_ON,R_.

The editable minimum, maximum, and step values of each parameter are reported in Table 1.

### 2.3. Readout Voltage Choice

**Figure 4:**
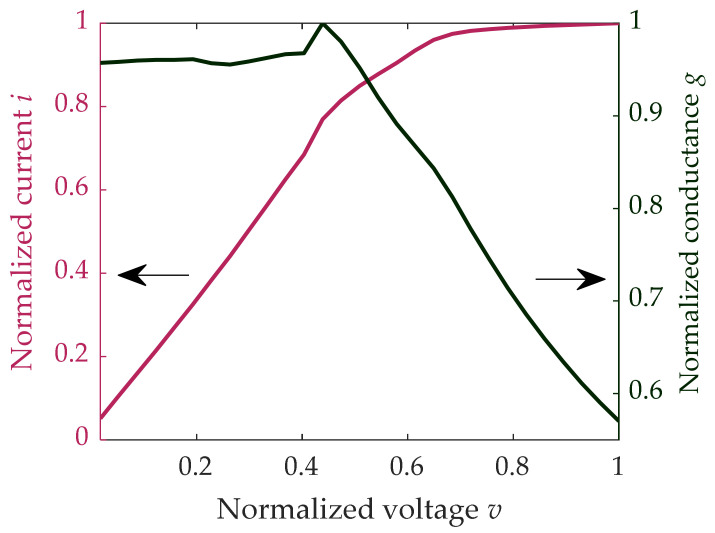
**Left** axes: typical normalized I-V characteristic obtained by averaging the currents of 5120 cells. **Right** axes: normalized cells mean conductance g = i/v.

The available hardware allows current measurements through the application to one or more cells of a readout voltage V_R_, ranging from 0 to V_R_^MAX^. The measured average *i*(*v*) characteristic of a group of PCM cells is depicted in Figure 4, where i is the cell current normalized to its maximum value, and v is defined as V_R_/V_R_^MAX^. 

The average normalized conductance g = i/v is nearly constant when V_R_ falls within [0–0.4] V_R_^MAX^; above V_R_ = 0.5 V_R_^MAX^, the voltage V_K_ applied to cells differs from V_R_ due to voltage drops of the transistors in the test chip readout circuitry. Therefore, due to test chip implementation, for the operation described in (2), V_k=1…N_ will be limited within the range [0–0.4] V_R_^MAX^. All measurements described hereafter are performed in the middle of that interval, namely, V_R_ = 0.25V_R_^MAX^ ≐ V_X_.

## 3. Results and Discussion

### 3.1. PCM Cell Characterization Using Single-SET Pulses

In this section, a characterization in terms of drift and noise is carried out. Cells were programmed through a single SET pulse. The following analyses were performed considering 5120 cells. Henceforth, conductances G are normalized to cell maximum conductance G^MAX^, and their currents I to I^MAX^ = G^MAX^V_X_, turning in cells normalized conductance g = G/G^MAX^ and normalized current i = I/I^MAX^, respectively. All the measurements, unless otherwise specified, were performed at room temperature.

#### 3.1.1. Noise

As previously observed, lattice imperfections and traps contribute to generate low-frequency noise, which affects the analog computation process [10,24,25,26]. Tests were performed in the following way: first, a start RESET pulse with A_R_ = 3A_R0_ and T_ON,R_ = T_ON,R0_ was applied to erase the previous state, followed by a SET pulse with T_ON,S_ = 2T_ON,S_, ΔI = ΔI_0_, ΔT = ΔT_0_. Four different values of A_S_ were considered: A_S0_, 1.5A_S0_, 2A_S0_, and 3A_S0_. To limit the time drift contribution, we performed measurements ≈12 h after the application of the SET pulse. Then, S_TOT_ = 188 current samples were collected for each cell at time intervals of 5 min t_i_. 

We evaluated the noise parameter *N*_%,*j*_ of the *j*-th cell as

(3) $$N_{\%,j}=\frac{100}{{\bar{g}}_{j}}\sqrt{\frac{1}{S_{TOT}-1}\overset{S_{TOT}}{\underset{i=1}{\sum }}{\left[g_{j}\left(t_{i}\right)-{\bar{g}}_{j}\right]}^{2}}$$

where *g_j_*(*t_i_*) is the *j*-th cell normalized conductance at time *t_i_*, and ${\bar{g}}_{j}$ is the time average of *g_j_*(*t_i_*).

**Figure 5:**
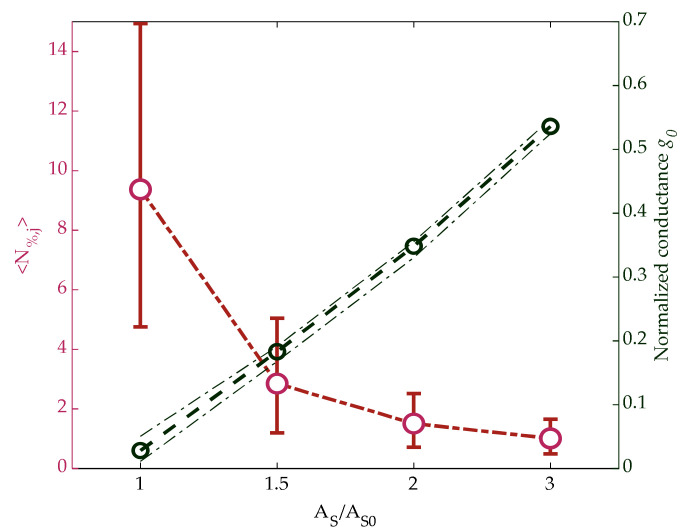
**Left**: ensemble average over all the tested cells of N_%,j_ defined in (3) vs. SET pulse amplitude. **Right**: normalized conductance averaged on both time and cells. Error bars and dashed lines represent the 10% and 90% limits of both distributions.

The ensemble average $\langle N_{\%,j}\rangle$ over all the tested cells is shown in Figure 5 (left) with red circles as a function of the amplitude A_S_, together with the indication of the 10% and 90% limits of the distribution. On the right vertical axis, the cell conductance averaged on both S_TOT_ = 188 time samples *t_i_*, and the 5120 measured cells are also shown, where the 10% and 90% limits of that distribution are depicted with dashed lines. The conductance was proportional to the SET amplitude, as expected, since a higher amplitude implies the crystallization of a wider cell volume. This leads to a reduction of noise, as its origin is mainly correlated to the lattice disordered structure of the amorphous phase [19,24,25].

**Figure 6:**
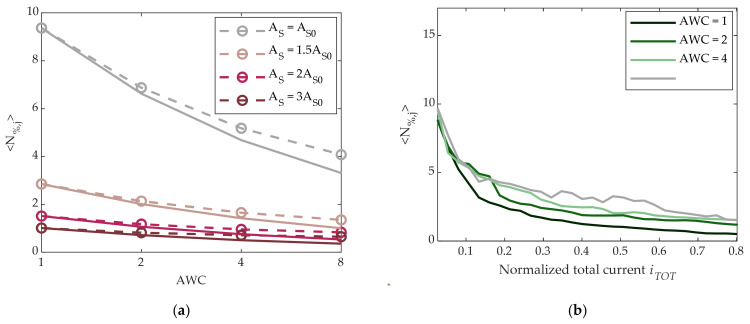
(**a**) Dotted lines: measured ensemble average $\langle N_{\%,j}\rangle$ of *N*_%,j_ defined in (3) vs. adjacent working cells (AWC) for different SET pulse amplitudes. Solid lines: theoretical $1/\sqrt{\mathrm{AWC}}$ noise behavior. (**b**) $\langle N_{\%,j}\rangle$ vs. normalized total current for different AWC values.

We then investigated the possibility of noise reduction by means of summing the current contributions of adjacent cells programmed in the same SET state. Measurements were performed with groups of 2, 4, or 8 adjacent working cells (AWC). To do so, we repeated previous measurements on a set of AWC × 5120 cells, and *N*_%_ was evaluated as in (3) but replacing *g*(*t_i_*) with the average of AWC cells for each sample time. Results are shown in Figure 6a as a function of AWC for different pulse amplitudes. If noise of different cells were totally uncorrelated, the curves would depend on AWC as $1/\sqrt{\mathrm{AWC}}$ (reported in the figure as solid lines). The differences can be ascribed to partial correlations induced by the measurement system.

As AWC > 1 for a given pulse amplitude results in an increase of power consumption, it is interesting to compare the cases AWC = 1 and AWC > 1 for the same normalized total current consumption. In Figure 6b, the ensemble average noise $\langle N_{\%,j}\rangle$ is reported as a function of the normalized total current for different AWC. It is clear that the AWC > 1 strategy is not convenient when power consumption is considered. In other words, for a given total current, a single cell achieves more noise reduction than several cells in parallel with lower conductance. For these reasons, the characterizations presented hereafter were performed with AWC = 1.

**Figure 7:**
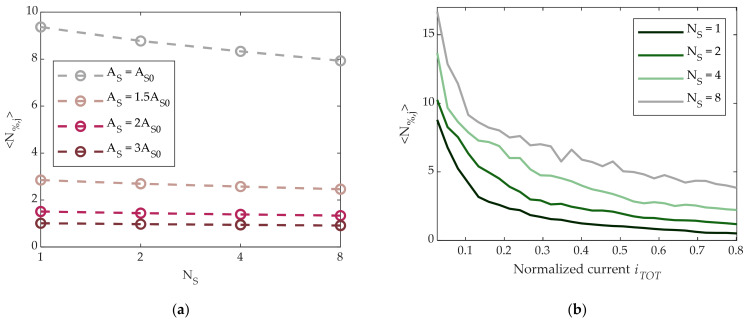
(**a**) Measured ensemble average $\langle N_{\%,j}\rangle$ of *N*_%,j_ defined in (3) vs. number N_S_ of samples in the averaging window for different SET amplitude pulses. (**b**) $\langle N_{\%,j}\rangle$ vs. normalized total current for different N_S_ values.

Finally, we explored the possibility to reduce noise through a time average operation. To this purpose, we repeated the previous measurements, and N_%_ was calculated replacing in (3) each *g*(*t_i_*) with the average over N_S_ consecutive samples equally separated in time by Δt = 5 min/N_S_, with N_S_ = 1, 2, 4, or 8. Results are shown in Figure 7a, where a slight reduction of noise is visible, in particular in the A_S0_-SET case. In analogy with the AWC strategy, it is necessary to consider the additional power consumption introduced by the N_S_-oversampling operation. *N*_%_ as a function of the normalized total current is shown in Figure 7b for the different values N_S_. It is seen that time average was not effective in terms of reducing noise for a given total current. This can be understood through taking into account the flicker nature of PCM cell noise [10,24,25,26], as time average operation is equivalent to a low-pass filter in the frequency domain.

A dependence of $\langle N_{\%,j}\rangle$ on SET pulse amplitude, AWC number, and time average, similar to the ones discussed in Figure 5, Figure 6 and Figure 7, was obtained varying T_ON,S_, ΔI/ΔT. To conclude, the most efficient strategy to reduce noise was the use of a single cell with a higher conductance for each matrix element.

#### 3.1.2. Time Drift

Short-term drift manifests itself as a slow but steady increase of the resistivity of the amorphous material [26]. The conductance *g*(*t*) drift has been shown to follow a power law $g\left(t\right)=g_{0}{\left(\frac{t}{t_{0}}\right)}^{-\gamma }$, where g_0_ is the initial conductance at arbitrary time *t*_0_, and *γ* is the drift coefficient, which is positive and cell-to-cell variable.

**Figure 8:**
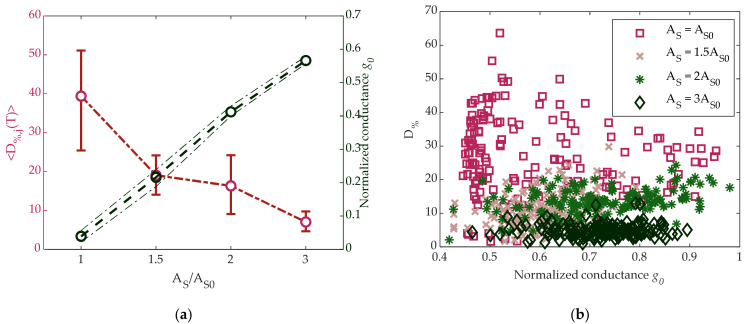
(**a**) Left: ensemble average of D_%_ defined in (4) with T = 14 h vs. SET pulse amplitude; right: mean value of the normalized conductance measured after the application of SET pulse. Error bars and dashed lines represent the 10% and 90% limits of both distributions. (**b**) D_%_ defined in (4) vs. normalized initial conductance g_0_ for different SET pulses amplitudes. Measures have been taken over a set of 960 cells.

In this work, instead of exploiting such a power law model, we evaluated drift in terms of relative conductance decrease *D*_%,*j*_ of the *j*-th cell as

(4) $$D_{\%,j}\left(t_{i}\right)=100\frac{g_{j,0}-g_{j}\left(t_{i}\right)}{g_{j,0}}$$

where *g_j_*(*t_i_*) is the *j*-th cell normalized conductance at time t_i_ and g_j,0_ its value measured 1 ms after the pulse application. We first investigated the effect of SET pulse amplitude on *D*_%_. To do so, we programmed 5120 cells in the same way explained in the previous paragraph, and then we monitored them for a time *t_i_* = *T* = 14 h at room temperature. The average $\langle D_{\%,j}\left(T\right)\rangle$ over all the tested cells as a function of the SET amplitude is shown in Figure 8a with red bullets as a function of the amplitude A_S_, and the indication of the 10% and 90% limits of the distribution are also shown. On the right vertical axis, the cell normalized mean conductance is plotted, where the two tiny dashed lines represent the 10% and 90% limits of the distribution. Results show that the increase of SET amplitude reduced cells drift below 8% for A_S_ = 3A_S0_. 

An additional result is reported in Figure 8b, where *D*_%_ for each cell is plotted vs. *g*_0_ for different pulse amplitudes. It can be observed that cells with the same initial conductance *g*_0_ had a lower drift when g_0_ was reached by applying a higher SET pulse.

### 3.2. PCM Cell Characterization Using Multiple Pulses 

In this section, we investigate the use of specific sequences of multiple current pulses to tune the cell conductance as close as possible to the desired level, while limiting noise, drift, and variability.

#### 3.2.1. Conductance Tunability

**Figure 9:**
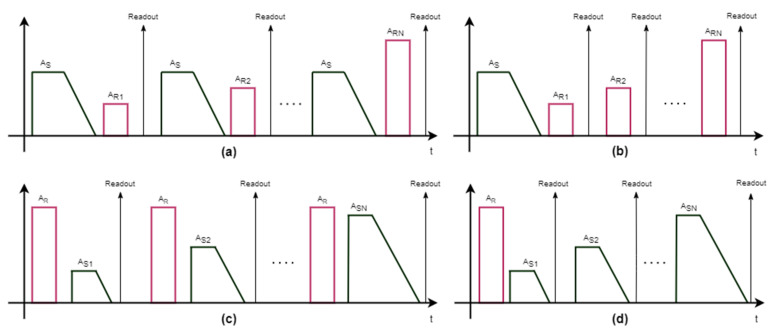
Analyzed programming sequences: (**a**) RESET single pulse (RSP); (**b**) RESET staircase (RSC); (**c**) SET single pulse (SSP); (**d**) SET staircase (SSC).

Cell reaction following the application of both a SET or a RESET pulse shows an uncertainty due to random amorphization and crystallization phenomena. The programming space is defined by the characteristic programming curve, which quantifies the change of the cell (normalized) conductance as a function of the programming pulse current. In the literature, two approaches have been proposed in order to program the cell resistance to an intermediate level: (a) partial-SET programming [27] and (b) partial-RESET programming [24,25]. In the first approach, the cell is first brought into the RESET state, and then a partial-SET programming pulse is applied so as to partially crystallize the active volume. In partial-RESET programming, the cell is first brought into the SET state, and then a partial-RESET pulse is applied in order to partially amorphize the active volume. On the basis of these two approaches, we experimented four different programming strategies and derived the corresponding programming curves. The adopted programming sequences are illustrated in Figure 9: (a) RESET single pulse programming (RSP); (b) RESET staircase programming (RSC); (c) SET single pulse programming (SSP); (d) SET staircase (SSC) programming.

In the RSP case (Figure 9a), first a SET pulse with A_S_ = 5A_S0_, T_ON,S_ = 2T_ON,S0_, ΔI = ΔI_0_, and ΔT = ΔT_0_ was applied, followed by a single partial-RESET pulse with a predetermined amplitude A_R_ and width T_ON,R_, and then, after 1 ms, a readout operation was performed. The above sequence was repeated with increasing values of A_R_ between A_R0_ and 4A_R0_ with steps of ≈A_R0_/10. In the RSC case (Figure 9b), a single start SET pulse with the same parameters mentioned above was applied only at the beginning, followed by a partial-RESET sequence identical to the one in the RSP case, with readout operations performed after each specific RESET pulse. 

**Figure 10:**
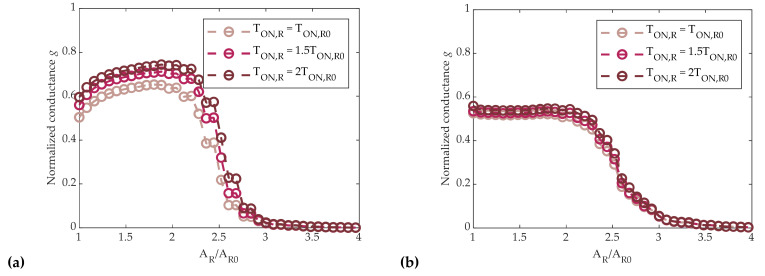
(**a**) RSP programming curves as a function of RESET pulse amplitude, with different T_ON,R_ values. The generic g(A_R,i_) represents cells normalized mean conductance after the application of a start SET pulse and a RESET pulse with amplitude A_R,i_. (**b**) RSC programming curves as a function of RESET pulse amplitude, with different T_ON,R_ values. The generic g(A_R,i_) represents cells normalized mean conductance after the application of a start SET pulse and a sequence of RESET pulses with amplitude from A_R0_ to A_R,i_.

Results of RSP and RSC are illustrated in Figure 10a,b, respectively, where the mean conductance of N_C_ = 5120 cells is plotted as a function of A_R_ for different values of T_ON,R_ (T_ON,R0_, 1.5T_ON,R0_, 2T_ON,R0_). The behavior of cells in RSP mode showed an initial increase of conductance, since small amplitude RESET pulses tend to be similar to a SET pulse. Then, when A_R_ > 2A_R0_, cell conductance began to decrease. This initial increase of the conductance value was absent in RSC mode. In both families of programming curves, the mean normalized conductance g slightly depended on T_ON,R_, whose value tended to increase the mean conductance of cells, as the RESET pulse was longer and tended to be more similar to a SET one. Furthermore, the programming curves for RSP or RSC were quite similar when A_R_ > 2A_R0_, with both being characterized by an abrupt decrease to a full RESET state.

For what concerns partial-SET programming, in the SSP case (Figure 9c), a start RESET pulse with A_R_ = 3A_R0_ and T_ON,R_ = 2T_ON,R0_ was applied, followed by a single partial-SET pulse and a readout operation. The sequence was repeated with A_S_ varying from A_S0_ to 4 A_S0_ in steps of ≈A_S0_/10. Adopted values of T_ON,S_ were T_ON,S0_, 1.5T_ON,S_, and 2T_ON,S0_. We chose ΔI = ΔI_0_ and ΔT = ΔT_0_ for all measurements. The SSC case (Figure 9d) was similar, but the start RESET pulse was applied only at the beginning. 

**Figure 11:**
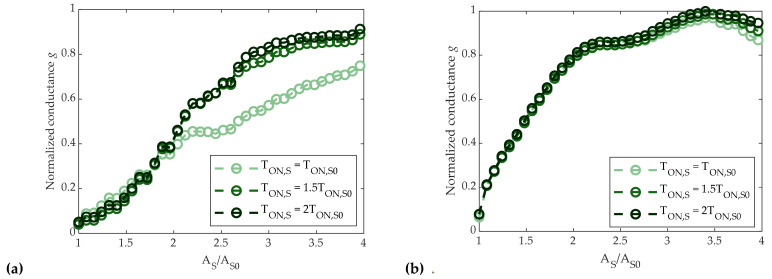
(**a**) SSP programming curves as a function of SET pulse amplitude, with different T_ON,S_ values. The generic g(A_S,i_) represents cells normalized mean conductance after the application of a start RESET pulse and a SET pulse with amplitude A_S,i_. (**b**) SSC programming curves as a function of SET pulse amplitude, with different T_ON,S_ values. The generic g(A_S,i_) represents cells normalized mean conductance after the application of a start RESET pulse and a sequence of SET pulses with amplitude from A_S0_ to A_S,i_.

As before, the mean conductance of 5120 cells was monitored. Results are reported in Figure 11a,b for the SSP and SSC cases, respectively. In these cases, the conductance was not significantly influenced by the value of T_ON,S_, except for the lowest value of T_ON,S_ in the SSP case. On the other hand, as opposed to the partial-RESET strategy, differences between the two sequences were indeed more visible—the SSC conductance tended to increase faster, reaching values above 90% of G^MAX^ with a lower SET amplitude (A_S_ = 2.2A_S0_), whereas the SSP conductance reached the same level only with a 3A_S0_–3.5A_S0_ SET pulse.

Comparing partial-RESET and partial-SET strategies, we can point out that RSP and RSC led to abrupt programming curves, whereas partial-SET programming allowed for a smoother control of the conductance by means of the SET amplitude. Thus, in view of a good conductance controllability, the partial-SET approach was found to be preferable. 

**Figure 12:**
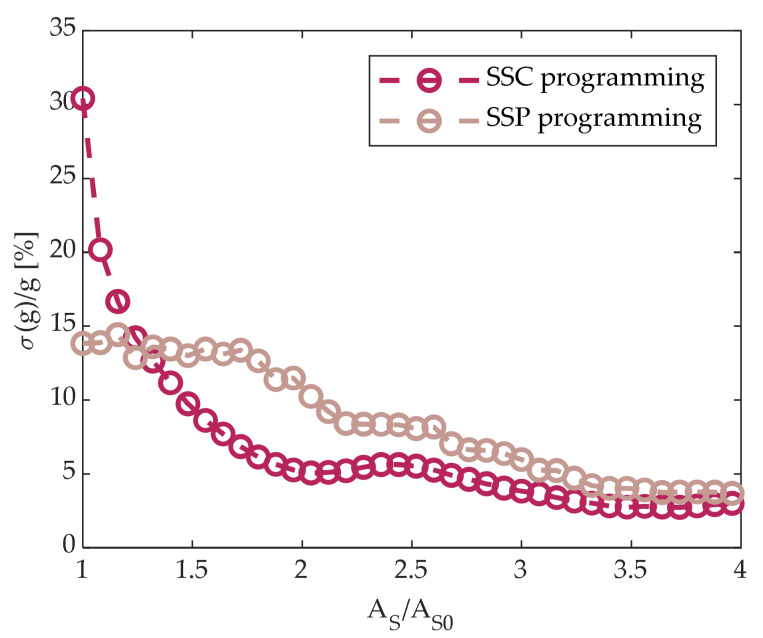
Normalized standard deviation σ(g)/g defined in (5) as a function of SET pulse amplitude for both SSP and SSC programming.

We also investigated the conductance spread induced by partial-SET programming evaluating the normalized conductance dispersion g_%_ at each SET amplitude step A_S,I_, defined as

(5) $$\frac{\sigma \left(g\right)}{g}\left(A_{S,i}\right)=\frac{100}{\langle g_{j}\left(A_{S,i}\right)\rangle }\sqrt{\frac{1}{N_{C}-1}\overset{N_{C}}{\underset{j=1}{\sum }}{\left[g_{j}\left(A_{S,i}\right)-\langle g_{j}\left(A_{S,i}\right)\rangle \right]}^{2}}$$

where the mean $\langle g_{j}\left(A_{S,i}\right)\rangle$ is calculated over the full set of *N_C_* = 5120 cells after the application of the *A_S_*_,*i*_-amplitude SET pulse. Results depicted in Figure 12 show that SSC programming led to a lower spread when A_S_ > 1.4A_S0_, where T_ON,S_ = 1.5T_ON,S0_. Additionally, SSP programming turned out to be more power-hungry, as it required a greater amount of RESET-applied pulses than the SSC programming to reach the same value of g.

**Figure 13:**
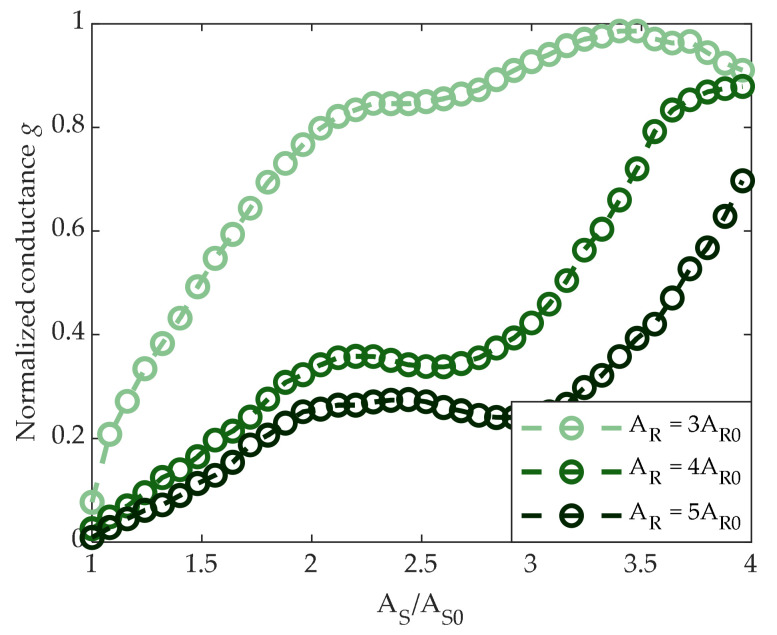
SSC programming curves as a function of SET pulse amplitude, with different values of the start RESET pulse amplitude.

We finally investigated the effect of the amplitude of the start RESET pulse on the SSC programming curve. Results are shown in Figure 13, where g vs. A_S_/A_S0_ for T_ON,S_ = 1.5 T_ON,S0_ is plotted for A_R_ = 3A_R0_, 4A_R0_, or 5A_R0_. It is seen that the conductance tended to increase more slowly for larger A_R_. In turn, larger SET pulse amplitudes were required to reach the same conductance level when A_R_ was larger_._ Therefore, the choice of the start RESET pulse amplitude played an important role in the programming curve; this property is exploited in the next paragraph.

To sum up, SSC programming seems to be the most convenient programming strategy, as it allows for both good conductance control and spread reduction.

#### 3.2.2. Drift-Induced Dispersion

The cell-to-cell conductance spread, which is initially determined by the finite resolution of the programming algorithm (see next section), tends to increase with time due to the cell-to-cell spread of the drift process described by the parameter *D*_%_ defined in (4). To investigate such drift spread, we characterized the *D*_%_ distribution, with the aim of optimizing the programming parameters in order to reduce its standard deviation σ(*D*_%_).

To this purpose, 5120 cells were programmed with an SSC strategy. After that, cell conductances were measured firstly after 14 h at room temperature (around 25 °C), and then after we heated the whole test chip to 150 °C for 48 h in a controlled climate chamber in order to emulate the maximum drift achievable by cells [28].

**Figure 14:**
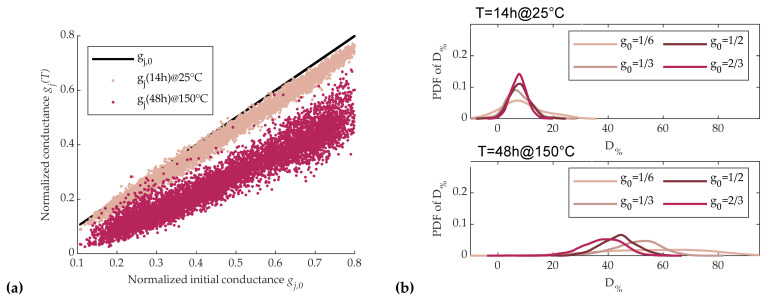
Effects of SSC sequence as described in Figure 9.d and 11.d with A_R_ = 3A_R0_. (**a**) Cells conductance as a function of the initial normalized conductance after 14 hours at room temperature, and after 48 hours at 150 °C. (**b**) Probability distribution of D_%_ obtained with the SSC programming sequence. Different curves refer to different target conductances with ± 10% tolerance.

Figure 14a shows the values of the measured normalized cell conductances as a function of their initial normalized conductance g_0_ after the first and the second time intervals. Among the resulting conductivities, a set of four increasing normalized conductivity values (*g*_0_ = 1/6, 1/3, 1/2, 2/3) was chosen. Figure 14b reports the probability distribution function (PDF) of D_%_ for such values of initial conductivity *g*_0_ ± 10%, where the top and the bottom plot refer to the first and the second measures, respectively. Results show that after 14 h, the mean value of *D*_%_ was quite independent of initial conductance value *g*_0_, while its dispersion tended to decrease for higher values of *g*_0_. After 48-h bake, both the mean value and dispersion of *D*_%_ were increased with respect to the first measure, and tended to decrease for higher values of *g*_0_, as can be observed also from Figure 14a.

**Figure 15:**
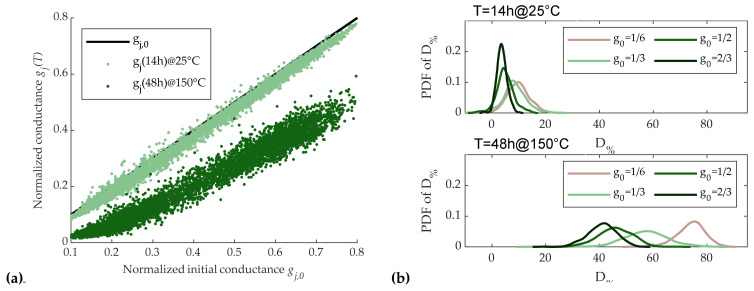
Effects of SSC sequence as in Figure 14 with the addition of an initial 5A_S0_ SET pulse and A_R_ = 5A_R0_. (**a**) Cells conductance as a function of the initial normalized conductance after 14 hours at room temperature, and after 48 hours at 150 °C. (**b**) Probability distribution of D_%_ obtained with the SSC programming sequence. Different curves refer to different target conductances with ± 10% tolerance.

The results on *D*_%_ in Section 3.2 show that a drift reduction was achievable using SET pulses of higher amplitude (see Figure 8b). Thus, as observed at the end of Section 3.2.1, we were able to use a higher-amplitude start RESET pulse in the SSC sequence to reach the same desired conductance with higher partial-SET pulses. Thus, we repeated the D_%_ dispersion analysis by increasing the start RESET pulse amplitude to 5A_R0_, instead of the 3A_R,0_ used for the results of Figure 14a,b. Moreover, as suggested in [23], an additional 5A_S0_ start SET pulse was applied before the start RESET pulse, with the aim of obtaining a more uniform cell initialization. The improvements induced by these choices are clearly visible in Figure 15a,b, which is to be compared with Figure 14a,b; for each *g*_0_, the average value of *D*_%_ was strongly reduced and the dispersion of *D*_%_ was quite reduced For the sake of completeness, we also performed measurements by varying the duration of the start SET pulse (T_ON,S_, 1.5T_ON,S_, and 2T_ON,S_), as well as those of the start RESET pulse (T_ON,R_, 1.5T_ON,R_, and 2T_ON,R_), but results did not significantly differ from those reported here.

The impact of high-amplitude SET pulses on endurance was not a severe constraint from the AIMC applications where a large amount of write cycle is not required.

## 4. A Programming Algorithm for AIMC

**Figure 16:**
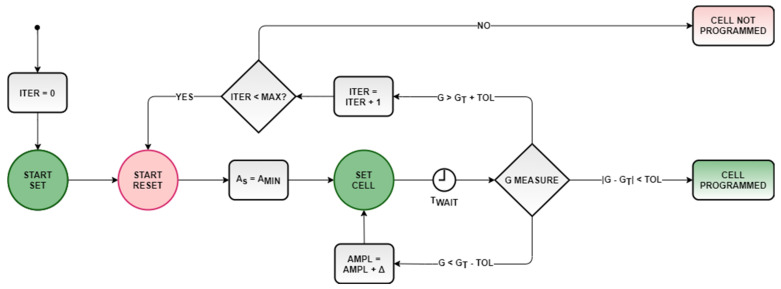
Proposed cells iterative programming algorithm. G indicates the measured cell conductance and G_T_ denotes the conductance target.

**Figure 17:**
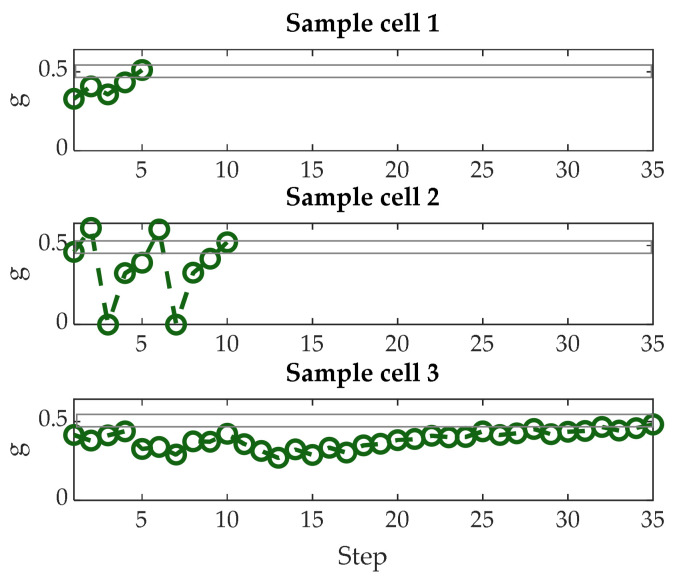
Typical evolution of the conductance of three sample cells during the programming sequence steps with the conductance target value set to 1/2 ± 10%. (1) Cell programmed in a few steps and only one iteration; (2) cell programmed in three iterations; (3) cell programmed with a long sequence of steps. The horizontal lines show conductance target ± 10%.

In this section, leveraging the characterizations described in the previous sections, we define an iterative programming algorithm, based on [23,24,25,26,27], aiming to set the cell conductance close to a desired value. The algorithm is outlined in Figure 16. Once the conductance target interval was defined, specifying the mean value and relative tolerance, the cell was first stimulated with the start SET and RESET pulses, as suggested by the results of the analysis discussed in the previous section. Then, the partial-SET SSC sequence (Figure 9d and Figure 11b) began with a minimum SET amplitude A_MIN_. After a predefined time, interval T_WAIT_, the cell current was read. If it fell within the target interval, the sequence was terminated. If the conductance was lower than the required limit, the cell was stimulated with a new SET pulse, with increased amplitude by a programmable step ΔA (see Figure 17, sample cells 1 and 3). If instead the conductance was above the upper limit, the whole process was restarted from the initial SET and RESET pulses (see Figure 17, sample cell 2). A maximum number of iterations ITER_MAX_ was defined—if the algorithm exceeded ITER_MAX_, the cell would be declared not programmed and would not be used in the final AIMC array.

Figure 17 shows the programming sequences relative to five sample cells, where the target was defined as 0.5G^MAX^ ± 10% tolerance. It must be noted that the definition of this tolerance set the maximum initial cell spread σ(g)/g defined in (5). A_MIN_ was set to 1.5A_S0_, ΔA to A_S0_/20, T_WAIT_ to 1 ms, and ITER_MAX_ = 100. In the same way, we programmed groups of N_PC_ = 128 cells with target *g*_0_ = 1/6, 1/3, 1/2, and 2/3, respectively. Table 2 summarizes the minimum, maximum, and average number of partial SET pulses required to program each cell, including possible restarted sequences. It can be noticed that the number of mean programming pulses increased with the conductance target, as we used the same A_MIN_ for every conductance goal. To improve the programming speed, A_MIN_ could be chosen in relation to the target level. An estimation of mean programming time is also provided in Table 2, which takes into account of the averaged durations of SET and RESET pulses only, assuming T_ON,S_ = 150 ns, as cell readout time is negligible in case the programming algorithm is performed on an embedded system [20]. Every cell was correctly programmed within the maximum 100 iterations.

**Figure 18:**
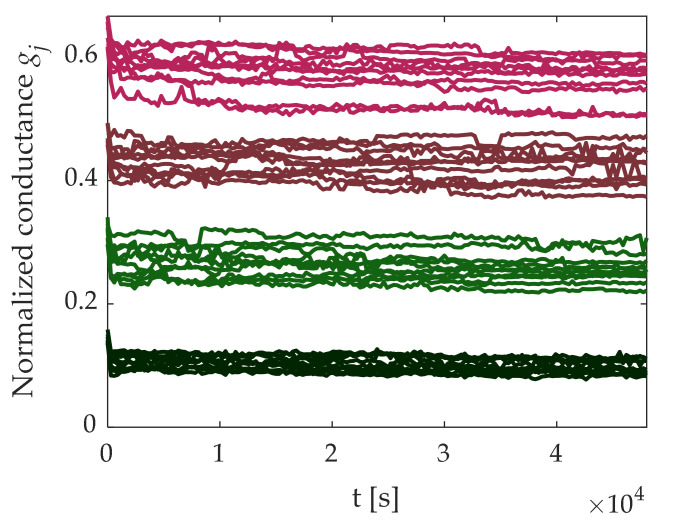
Programmed cell conductance behavior monitored for 14 h. Only 10 cells for each group are plotted. Initial normalized conductance target values were 1/6, 1/3, 1/2, and 2/3.

**Figure 19:**
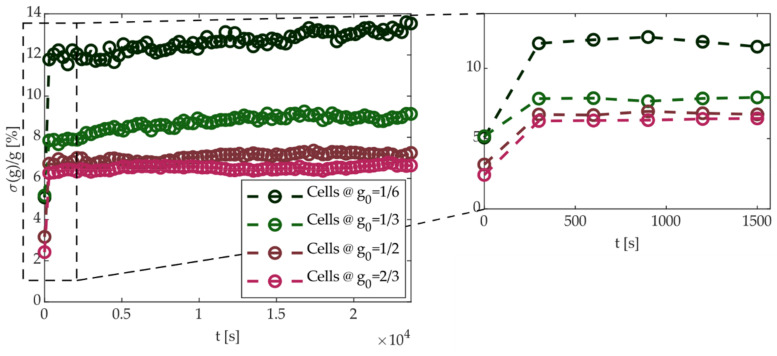
Cell conductance spread σ(g)/g defined in (6) vs. time. A zoom on the first six measures is shown the effect of drift on the initial spread set by the proposed programming algorithm.

Then, the programmed cell conductance was monitored for ≈14 h (160 samples with 5-min steps), whose time evolution is depicted in Figure 18. It must be noticed that four different levels of conductance were distinguishable in the whole observation time interval. For each programmed group of N_PC_ = 128 cells, we calculated the conductance spread defined as

(6) $$\frac{\sigma \left(g\right)}{g}\left(t_{i}\right)=\frac{100}{\langle g_{j}\left(t_{i}\right)\rangle }\sqrt{\frac{1}{N_{PC}-1}\overset{N_{PC}}{\underset{j=1}{\sum }}{\left[g_{j}\left(t_{i}\right)-\langle g_{j}\left(t_{i}\right)\rangle \right]}^{2}}$$

with results being reported in Figure 19. The initial value was under 6% in all cases (5.08%, 5.17%, 3.16%, and 2.42% for *g*_0_ = 1/6, 1/3, 1/2, and 2/3, respectively), lower than the target tolerance ± 10%. Then, due to the random conductance drift, GS_%_ tended to increase in the first readout interval (5 min). After that time, spread did not change significantly, suggesting that the effect of drift was appreciable mostly in the first 5 min (or less). Moreover, cells with higher conductance showed a lower and less variable spread, consistent with the previous analysis (see Figure 15).

**Figure 20:**
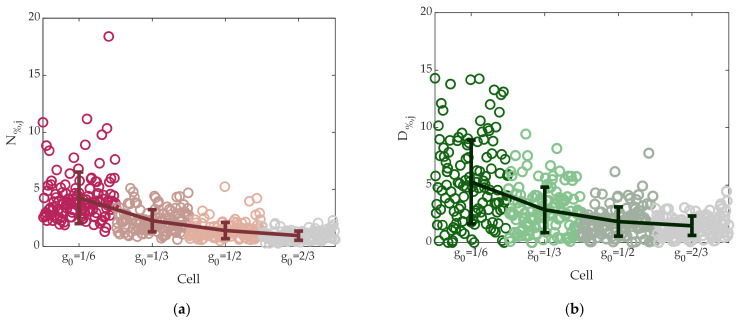
(**a**) N_%,j_ defined in (3) of the 512 programmed cells. Circles represent noise of single cells. Error bars indicate noise mean value for the four conductance target levels, together with the 10% and 90% limits of the distribution. (**b**) D_%,j_ defined in (4) of the 512 programmed cells. Circles represent drift of single cells. Error bars indicates noise mean value for the four conductance target levels, together with the 10% and 90% limits of the distribution.

Noise was evaluated through taking the last 120 samples occurring after 4 h from the application of the programming sequence to neglect initial strong drift effects. Results are shown in Figure 20a with circles, where N% defined in (3) for each of the 512 cells is reported. Cells with the lowest conductance were characterized by N_%_ in the 2–10% range (except for two cells); the lowest noise, less than 2%, was achieved by the cells with the highest conductance *g*_0_ = 2/3.

Finally, *D*_%_ defined in (4) is shown in Figure 20b with circles. Results showed a decrease of conductance loss for higher-conductance, and *D*_%_ was lower than 10% for all cells except for the ones with the lowest conductance levels. This is a key feature of SSC programming strategy combined with the adoption of start SET and start RESET pulses.

Solid lines in Figure 20a,b report the ensemble average $\langle N_{\%,j}\rangle$ and $\langle D_{\%,j}\rangle$ over all the 512 tested cells with circles as a function of the conductance target, together with the indication of the 10% and 90% limits of the distributions.

The present study can represent a valid basis to define optimal programming algorithms for AIMC applications based on PCM, provided some improvements are introduced. For example, the total time necessary for the full programming sequences can be reduced by starting from amplitude pulse levels that are functions of the target conductance. However, it should be noted that AIMC applications do not require particularly fast and frequent write cycles.

## 5. Conclusions

In this work, a characterization of PCM cells for AIMC applications was carried out. Cell non-idealities, i.e., low-frequency noise, time drift, and conductance spread, lead to inaccuracies that affect the computation process accomplished by the memory array. Proper cell programming sequences to mitigate these undesired effects are proposed. In particular, higher applied SET amplitude pulses lead to better performance in terms of noise. In addition, results have shown that, for a given target conductance, a single cell achieves more noise reduction than several cells in parallel, each having lower conductance. Moreover, drift is reduced when high SET amplitude pulses are employed. The SSC programming strategy ensures better results in terms of cell spread and initial conductance control. Moreover, the application of large start SET and RESET pulses at the beginning of the programming sequence achieves a better cells dispersion performance.

As an example of application of the above considerations, the results of programming 512 cells with four different conductance levels are shown. The cell conductances were monitored up to 14 h after the application of the programming procedure. For all memory cells, the measured conductance spread was under 14% and the relative drift under 15%, with the relative noise less than 9% for 90% of cells.

We believe that the present study can represent a valid basis to define optimal programming algorithms for AIMC applications based on PCM, which partially circumvent the intrinsic non-idealities of PCM cells.

## Figures and Tables

**Table 1:** Configurable parameters of SET and RESET pulses.

Parameter	Minimum	Maximum	Resolution	Order of Magnitude
A_S_^1^	A_S0_	≈6 A_S0_	≈A_S0_/10	10–100 μA
T_ON,S_	T_ON,S0_	2 T_ON,S0_	T_ON,S0_/2	100 ns
ΔI	ΔI_0_	2 ΔI_0_	ΔI_0_	10 μA
ΔT	ΔT_0_	2 ΔT_0_	ΔT_0_/2	10 ns
A_R_^1^	A_R0_	≈6 A_R0_	≈A_R0_/10	10–100 μA
T_ON,R_	T_ON,R0_	2 T_ON,R0_	T_ON,R0_/10	10 ns

^1^ Amplitude parameters A_S_ and A_R_ are slightly variable as they depend on the readout conversion chain calibration.

**Table 2:** Required number of steps for cells programming and estimated programming times.

Normalized Conductance Target	Number of Steps			Estimated Programming Time	
	Minimum	Maximum	Mean	Mean	Maximum
1/6	2	20	6	900 ns	3 µs
1/3	2	45	10	1.5 µs	6.75 µs
1/2	2	64	22	3.3 µs	9.6 µs
2/3	3	95	36	5.4 µs	9.75 µs

## References

[1] Burr G.W., Kurdi B.N., Scott J.C., Lam C.H., Gopalakrishnan K., Shenoy R.S. (2008). Overview of candidate device technologies for storage-class memory. IBM J. Res. Dev..

[2] Annunziata R., Zuliani P., Borghi M., De Sandre G., Scotti L., Prelini C., Tosi M., Tortorelli I., Pellizzer F. Phase Change Memory technology for embedded non volatile memory applications for 90nm and beyond. Proceedings of the 2009 IEEE International Electron Devices Meeting (IEDM).

[3] Raoux S., Burr G.W., Breitwisch M.J., Rettner C.T., Chen Y.-C., Shelby R.M., Salinga M., Krebs D., Chen S.-H., Lung H.-L. (2008). Phase-change random access memory: A scalable technology. IBM J. Res. Dev..

[4] De Sandre G., Bettini L., Pirola A., Marmonier L., Pasotti M., Borghi M., Mattavelli P., Zuliani P., Scotti L., Mastracchio G. A 90nm 4Mb embedded phase-change memory with 1.2V 12ns read access time and 1MB/s write throughput. Proceedings of the 2010 IEEE International Solid-State Circuits Conference-(ISSCC).

[5] Ielmini D., Pedretti G. (2020). Device and Circuit Architectures for In-Memory Computing. Adv. Intell. Syst..

[6] Sun Z., Pedretti G., Ambrosi E., Bricalli A., Wang W., Ielmini D. (2019). Solving matrix equations in one step with cross-point resistive arrays. Proc. Natl. Acad. Sci. USA.

[7] Joshi V., Le Gallo M., Haefeli S., Boybat I., Nandakumar S.R., Piveteau C., Dazzi M., Rajendran B., Sebastian A., Eleftheriou E. (2020). Accurate deep neural network inference using computational phase-change memory. Nat. Commun..

[8] Sebastian A., Le Gallo M., Burr G.W., Kim S., BrightSky M., Eleftheriou E. (2018). Tutorial: Brain-inspired computing using phase-change memory devices. J. Appl. Phys..

[9] Cristiano G., Giordano M., Ambrogio S., Romero L.P., Cheng C., Narayanan P., Tsai H., Shelby R.M., Burr G.W. (2018). Perspective on training fully connected networks with resistive memories: Device requirements for multiple conductances of varying significance. J. Appl. Phys..

[10] Tuma T., Pantazi A., Le Gallo M., Sebastian A., Eleftheriou E. (2016). Stochastic phase-change neurons. Nat. Nanotechnol..

[11] Burr G.W., Shelby R.M., Sebastian A., Kim S., Kim S., Sidler S., Virwani K., Ishii M., Narayanan P., Fumarola A. (2017). Neuromorphic computing using non-volatile memory. Adv. Physics X.

[12] Ielmini D., Ambrogio S. (2019). Emerging neuromorphic devices. Nanotechnolgy.

[13] Sze V., Chen Y.-H., Yang T.-J., Emer J.S. (2017). Efficient Processing of Deep Neural Networks: A Tutorial and Survey. Proc. IEEE.

[14] Ou Q.-F., Xiong B.-S., Yu L., Wen J., Wang L., Tong Y. (2020). In-Memory Logic Operations and Neuromorphic Computing in Non-Volatile Random Access Memory. Materials.

[15] Milo V., Malavena G., Compagnoni C.M., Ielmini D. (2020). Memristive and CMOS Devices for Neuromorphic Computing. Materials.

[16] Park J. (2020). Neuromorphic Computing Using Emerging Synaptic Devices: A Retrospective Summary and an Outlook. Electronics.

[17] Kersting B., Ovuka V., Jonnalagadda V.P., Sousa M., Bragaglia V., Sarwat S.G., Le Gallo M., Salinga M., Sebastian A. (2020). State dependence and temporal evolution of resistance in projected phase change memory. Sci. Rep..

[18] Pirovano A., Lacaita A., Pellizzer F., Kostylev S., Benvenuti A., Bez R. (2004). Low-field amorphous state resistance and threshold voltage drift in chalcogenide materials. IEEE Trans. Electron Devices.

[19] Papandreou N., Pozidis H., Pantazi A., Sebastian A., Breitwisch M.J., Lam C.H., Eleftheriou E. Programming algorithms for multilevel phase-change memory. Proceedings of the 2011 IEEE International Symposium of Circuits and Systems (ISCAS); Institute of Electrical and Electronics Engineers (IEEE).

[20] Carissimi M., Zurla R., Auricchio C., Calvetti E., Capecchi L., Croce L., Zanchi S., Rana V., Mishra P., Mukherjee R. 2-Mb Embedded Phase Change Memory With 16-ns Read Access Time and 5-Mb/s Write Throughput in 90-nm BCD Technology for Automotive Applications. Proceedings of the ESSCIRC 2019-IEEE 45th European Solid State Circuits Conference (ESSCIRC); Institute of Electrical and Electronics Engineers (IEEE).

[21] Pasotti M., Zurla R., Carissimi M., Auricchio C., Brambilla D., Calvetti E., Capecchi L., Croce L., Gallinari D., Mazzaglia C. (2018). A 32-KB ePCM for Real-Time Data Processing in Automotive and Smart Power Applications. IEEE J. Solid-State Circuits.

[22] Nirschl T., Chen C.-F., Joseph E., Lamorey M., Cheek R., Chen S.-H., Zaidi S., Raoux S., Chen Y., Zhu Y. Write Strategies for 2 and 4-bit Multi-Level Phase-Change Memory. Proceedings of the 2007 IEEE International Electron Devices Meeting.

[23] Bedeschi F., Fackenthal R., Resta C., Donze E.M., Jagasivamani M., Buda E.C., Pellizzer F., Chow D.W., Cabrini A., Calvi G.M.A. (2008). A Bipolar-Selected Phase Change Memory Featuring Multi-Level Cell Storage. IEEE J. Solid-State Circuits.

[24] Cabrini A., Braga S., Manetto A., Torelli G. Voltage-Driven Multilevel Programming in Phase Change Memories. Proceedings of the 2009 IEEE International Workshop on Memory Technology, Design, and Testing.

[25] Braga S., Sanasi A., Cabrini A., Torelli G. (2010). Voltage-Driven Partial-RESET Multilevel Programming in Phase-Change Memories. IEEE Trans. Electron Devices.

[26] Ielmini D., Lacaita A.L., Mantegazza D. (2007). Recovery and Drift Dynamics of Resistance and Threshold Voltages in Phase-Change Memories. IEEE Trans. Electron Devices.

[27] Zhang Y., Feng J., Zhang Y., Zhang Z., Lin Y., Tang T., Cai B., Chen B. (2007). Multi-bit storage in reset process of Phase Change Access Memory (PRAM). Phys. Status Solidi (RRL) Rapid Res. Lett..

[28] Volpe F.G., Cabrini A., Pasotti M., Torelli G. Drift induced rigid current shift in Ge-Rich GST Phase Change Memories in Low Resistance State. Proceedings of the 2019 26th IEEE International Conference on Electronics, Circuits and Systems (ICECS); Institute of Electrical and Electronics Engineers (IEEE).

